# An Open-Source Scale Model Platform for Teaching Autonomous Vehicle Technologies

**DOI:** 10.3390/s21113850

**Published:** 2021-06-02

**Authors:** Bastien Vincke, Sergio Rodriguez Florez, Pascal Aubert

**Affiliations:** 1 Department of Metrology and Applied Physics, University Institute of Technology of Orsay, 91405 Orsay, France; sergio.rodriguez@u-psud.fr (S.R.F.); pascal.aubert@universite-paris-saclay.fr (P.A.); 2 SATIE Laboratory CNRS Joint Research Unit, UMR 8029, Paris-Saclay University, 91190 Gif-sur-Yvette, France; 3 C2N Laboratory CNRS Joint Research Unit, UMR 9001, Paris-Saclay University, 91190 Gif-sur-Yvette, France

**Keywords:** scale-model vehicle, embedded systems, technological teaching

## Abstract

Emerging technologies in the context of Autonomous Vehicles (AV) have drastically evolved the industry’s qualification requirements. AVs incorporate complex perception and control systems. Teaching the associated skills that are necessary for the analysis of such systems becomes a very difficult process and existing solutions do not facilitate learning. In this study, our efforts are devoted to proposingan open-source scale model vehicle platform that is designed for teaching the fundamental concepts of autonomous vehicles technologies that are adapted to undergraduate and technical students. The proposed platform is as realistic as possible in order to present and address all of the fundamental concepts that are associated with AV. It includes all on-board components of a stand-alone system, including low and high level functions. Such functionalities are detailed and a proof of concept prototype is presented. A set of experiments is carried out, and the results obtained using this prototype validate the usability of the model for the analysis of time- and energy-constrained systems, as well as distributed embedded perception systems.

## 1. Introduction

In recent years, the accelerated introduction of robotic functions into Intelligent Transportation Systems (ITS) makes associated socioeconomic sectors evolve and rapidly change. The automotive sector is moving towards the massive deployment of Advanced Driver Assistance Systems (ADAS), the introduction of Autonomous Vehicles (AV), and energy transition (i.e., electromobility). The field of AV is a very active one, and many innovations emerge every month. Many production vehicles are equipped with SAE Level 2 driver assistance functions. Further SAE Levels, such as 3 to 5, are still under development, and their introduction is expected in a near future. Tesla is undoubtedly the best-known brand in this field, which offers a very high level of autonomy in its cars. Similarly, Google is very active in this area, and it offers many innovations. However, very little information on electronic architecture is available on these models. Tesla is known to embed NVidia GPUs in their vehicles to perform processing. Google remains relatively discreet regarding the architectures used. Therefore, we have chosen to give particular focus to the experimental vehicles dedicated to AV related research.

AV research is essentially structured in two folds: data-driven research and experimental platform design. Data-driven research usually exploits referenced databases to develop new perception and inference algorithms. The experimental platform design is well-suited for studying navigation methods and evaluating perception under thorough use-case scenarios.

KITTI introduced a well-known example of data driven research where an experimental vehicle was devoted to the creation of sensor databases [1]. This platform was employed to collect a great amount of data that is now a reference to the research community. It carries two pairs of stereoscopic cameras, a Velodyne LiDAR and an GNSSS/IMU unit. All of the sensor data are recorded on a central computer. The architecture is a centralized high level architecture that only considers perception functions. Other experimental vehicles, such as Broggi et al. [2], were intended for extensive testing perception and control functionalities under thorough conditions over a 13,000 Km long trajectory. The robotcar platform [3] was recently developed from a Nissan LEAF. It carries several laser rangefinders and cameras. The data are processed by a central computer that collects and processes it. The platform is strongly based on the use of laser rangefinders to produce 3D maps. A dataset from this platform has been made publicly available.

Taking all of these experimental platforms and their wide functional range into consideration, a trade-off design should meet the architecture of a classical vehicle and the innovative sensing properties of an autonomous prototype. The architecture of a classic vehicle is very well known. It is based on the use of the CAN network, which has many advantages for this use. Regarding autonomous vehicles, there are no standards, since they are still under development. For us, an AV architecture can be structured into two parts.

The low level architecture is composed of the Engine Control Unit (ECU) in a commercial car. It manages all the functions of the car, such as the engine driver or the steering. It mainly operates under real-time constraints (with low latency). The measurement analysis is mostly close to the sensor. Low level architecture only includes low data flow.

Regarding the high level, it incorporates low real-time constraints (small latency order of 10 ms). It is well-suited for high data flow and data fusion approaches. It is clear that several types of sensors are commonly used: camera, laser telemeter, GPS, and inertial control unit that we do not find in a classic car. One or more processing units are added on these high level sensors in order to process the data and then send the instructions to the low level units. Therefore, our platform will include these two levels of processing.

Unfortunately, in order to face those new industrial skill requirements, very few autonomous platform alternatives exist, and none of them are devoted to undergraduate students. There are many robotic platforms for addressing part of the problem. However, these platforms are not very representative of a full-scale vehicle. They are mostly based on a centralized electronic architecture (very often mono-processor), and they do not differentiate high and low level functionalities.

We propose a robotized platform that is representative of today’s autonomous cars. It is composed of a mechanical chassis of a radio-controlled 1:10 scale car model. This mechanical base allows us to obtain a non-holonomic motion, like that for a full-scale car: control of the steering angle and wheel speed. For the embedded electronic architecture, a CAN network distributed node-based communication system was adopted as the one deployed in the automotive industry. The use of communicating nodes allows us to structure all of the complex functions into unitary functional blocks. Moreover, it allows the students to easily work on the scale-model and make the system adaptable when integrating new functionalities. For the high level part, we have integrated two processors that are powerful enough to handle large data flows. Indeed, we chose to embed widely used sensors in autonomous cars (stereoscopic cameras and laser rangefinders). Thus, it is possible to embed the algorithms in real time on the platform to evaluate them. To this end, this paper also presents, at a glance, the results of a Simultaneous Localization and Mapping (SLAM) approach and a Computer Vision-based object detector utilizing onboard sensors. Our platform wants to be as realistic as possible in order to train our students. However, it must remain relatively flexible and accessible, so that students can continue to develop our model.

Thus, the contributions of this work are:

- A scale-model design integrating a realistic electronic and communication bus architecture as full-scale AV solutions. The proposed design integrates sensors and communication systems that are well-suited for teaching purposes.
- An experimental validation of the onboard perception of the proposed scale-model design through the use of state-of-art approaches: Ccomputer Vision-based object detection and a SLAM algorithm.
- A publicly available dataset of laser rangefinder experiment.

To the best of the authors’ knowledge, the proposed design is innovative and original, since it efficiently combines high and low-level functionalities in a scale model vehicle. This architecture concept is a fundamental basis of an embedded real-time robotics system, and it constitutes key training support for engineering students. This design can certainly also facilitate the knowledge transfer between AV research and teaching programs.

In Section 2, the remaining of this paper presents a synthesis of state-of-the-art robotics teaching solutions and scale-model vehicles. Subsequently, Section 3 describes the context and objectives that are addressed by our scale-model vehicle design. Section 4 is devoted to formally detailing the scale-model architecture, electronics components, and onboard sensing modalities. The proposed platform is then experimentally validated by a student in Section 5, and we propose a new assignement in Section 6. Finally, conclusions and perspectives are drawn in Section 7 and Section 8.

## 2. Related Works

Technological and practical teaching in the field of AVs require the use of a versatile, flexible, and evolving robotic platform that is similar enough to a full-scale car regarding its motion (i.e., dynamics), its on-board electronic architecture, and its perception modalities. Such teaching requirements have been addressed in the past years attempting to grant access to students to a didactic material where fundamental concepts of the fields of metrology, instrumentation, electronics, and mobile robotics can be illustrated, taught, and experimentally validated. Hereafter, an outlook of existing robotics platforms is developed as a first attempt to identify their strengths and weaknesses. Further, special focus is given to recent scale car models (Table 1).

### 2.1. Robotics Platforms

Some of the most studied and developed robots are Epuck and Thymio, which were introduced in Mondada et al. [4] and [6,7], respectively. Epuck first emerged based on the concept of a sharing community consolidated by a common robotic system. This concept particularly helps to stimulate exchanges in the robotics research community. Epuck benefits from a compact body block design that integrates a large range of sensors (proximity, inertial, camera, and line detectors) in a differential robot motion structure. Becase Epuck includes a dsPic embedded processing unit, only low-complexity data processing can be deployed in this type of solution. Because of its relative high cost, Epuck was rarely affordable for undergraduate classes. Under this consideration, Thymio was introduced as a low-cost robot integrating obstacle detection sensory, line following sensors, and inertial sensors. The numerous robotic platforms that are available today cover all of the needs for the training of students in embedded electronics.

### 2.2. Scale Vehicle-Like Platforms

Several platforms have been designed to enable similar use conditions to those of an autonomous car. Some among them [16] were intended for academic robotic contests.

Audi proposed one of the first AV competitions in 2018. They designed a fully equipped 1:8 platform, which was loaned to each team. The vehicle is equipped with a front and a reverse camera, a laser scanner, a ultrasound sensors, and an inertial measurement unit. On-board, they chose to include a very important computing power and a GPU card in massive optimized data processing. This platform was intended for allowing teams to focus on high-level perception and navigation strategies and algorithms.

More recently, Amazon has developed a robotics competition called “AWS DeepRacer” [14]. It includes an 1:18 scale autonomous racing car that is designed to enable thorough evaluation of artificial intelligence navigation models by racing on a real scale track. The platform includes a stereoscopic camera, a scanning laser rangefinder, and an IMU. On the processing side, it embeds an Atom processor card, and it is compatible with ROS and Intel OpenVINO. The platform is dedicated to high level algorithms. Its compactness is its main advantage. However, this is also its main flaw, because it is absolutely not modifiable.

Beyond competitions, some platforms have been designed as research and teaching platforms. The open-source MIT RACECAR [13] is based on a 1:10 scale model, and it is based on the principles of a real moving car. It embeds state of art sensors: laser range finder, stereo camera, depth camera, and an IMU. The platform has an important computing power (NVIDIA Jetson), and it is focused on high level functions. Thus, the low level has been simplified as much as possible. Another open-source platform is the F1TENTH [16]. One can build it yourself by following the instructions provided. The car is built around a model chassis, embedded conventional sensors (laser rangefinders, RGBD camera), and conventional microcontroller cards (NVIDIA Jetson TX2). The car is dedicated to speed racing. It integrates a very good quality Electric Speed Controller allowing fine adjustments regarding the control of the vehicle.

The platform Quanser Q-Car [15] has been very recently proposed. It is an open-architecture scale-model vehicle. It was built to be accessible to students while including all the components of today’s autonomous car. At the sensor level, it embeds a LiDAR, an RGBD camera, two side cameras, microphones, odometers, and an inertial control unit. In addition, it is equipped with a custom-made motherboard, including a NVIDIA Jetson TX2 processor. This type of product is mainly differentiated by its industrial manufacturing quality.

Many of existing robotic platforms are adapted to the study of low level functionalities, such as the study of sensors, interfacing, and embedded programming. These robotic platforms do not have vehicle dynamics. On the other hand, platforms that are based on radio-controlled vehicle chassis almost satisfy teaching needs regarding low level functionalities. However, exiting platforms limit their application to high level functions. They focus on high level sensors (camera and laser) by simplifying the low level as much as possible and, therefore, are especially not representative of the electronic architecture of an autonomous car. Table 1 summarizes all of the platforms presented.

The advantage of our proposed platform in comparison to the existing ones can be emphasized, as follows:

- the platform is completely customizable and it can be adapted to the teaching needs;
- inspired from a real car, our platform has a distributed electronic architecture and a CAN network to communicate between the processing different nodes;
- the platform has pre-configured nodes, including the technologies used by an autonomous cars;
- thanks to its modular architecture, the platform allows working at different levels (sensors, electronics, and algorithms) while taking advantage of the rest of the car; and,
- structural elements of the proposed platform design and a sample dataset are publicly available, which enable replication, validation, or use in teaching applications.

## 3. Teaching Autonomous Vehicle Skills: Requirements and Objectives

### 3.1. Industrial Skill Requirements

The automotive world has been working, for several years, on the theme of the autonomous car. Every year, we see new functionalities, new lines of research, and new technologies that are associated with the autonomous car.

However, the world of education is struggling to train its students in such a dynamic field. In France, there is very little training regarding vehicles and even less concerning autonomous vehicles. Most of the training courses are devoted to training on vehicle propulsion and not on the functions of autonomous vehicles and ADAS.

### 3.2. Pedagogical Objectives

The pedagogical objectives that led us to designing our platform are numerous. We wanted to be able to study all the fields that are related to the autonomous automobile. The fields range from low level embedded electronics (sensors, interfacing) to the study of high level algorithms (artificial intelligence).

The main goal is to enable training future engineers who will work on autonomous platforms at both low and high levels. Therefore, it was necessary to have a platform that was as realistic as possible.

### 3.3. Pedagogical Skills and Proficiency

By using the platform, it will be possible to acquire many technical skills, but also train in teamwork.

#### 3.3.1. Technical Skills

We could address a wide range of technical skills while using the platform. The first level concerns the study of sensors and the metrology that is associated with these sensors. Indeed, it is very important to take the measurement uncertainties related to sensors into account, and this issue can be easily highlighted on this platform.

It will be possible to study the communication buses that are embedded on the platform. The platform is an excellent example of the use of the CAN network. Within a sensor node, it will also be possible to study the I2C or SPI bus.

It is possible to study low level automatic functions, such as speed control of the car. This servoing could be implemented at several levels to study the interest of integrating the processing as close as possible to the sensor or the actuator.

Higher level sensors could be used to deal with algorithmic problems, such as object, line, target recognition, or any artificial intelligence problem. The platform can be used to implement high level algorithms (like SLAM and DATMO) or, more generally, data fusion algorithms. Finally, it is also an excellent platform for testing the navigation and path planning algorithms.

#### 3.3.2. Student Skills

The platform was designed to promote teamwork. Each student or pair of students could work on a part of the platform and share their work. The platform acts more like a project work over several weeks. Indeed, the platform requires a significant amount of time to get used to it in order to make use of its complete potential. If the working time is reduced to less than a day, then it is possible to study a function of the car separately. For example, we could study the CAN network or a camera-based perception function.

### 3.4. Students Profile

The platform has been designed to be usable for all levels of high school students.

It is possible to use the platform using only the very high level functions and to only program one of the microcontrollers. This is perfectly sufficient for discovering the problems that are related to embedded systems and AV. We apply a processor used very commonly today (Atmega368p from Microchip, Chandler, AZ, USA). The use of this processor made us compatible with high level languages, like mBlock or Scratch. This allows us to use the platform to discover embedded systems.

It is then possible to discover the electronic part of the platform. It seems possible to discover some functions with an average level in electronics. Indeed, the car’s functions are separated in each function block. Therefore, it is possible to separately study the functions. To us, this discovery seems to be suitable for students between 16 and 20 years old. It is quite possible to create new functions in a simple way and interface its module with the CAN network of the car.

High level functions (camera, LiDAR, and indoor GPS) can be used by two types of students. The students can use already programmed high-level functions and just create a suitable driving behavior. This type of work can be done as soon as the student is able to understand the high and low level functions of the car. It is also possible to develop one’s own algorithms from the raw data of the car. For example, it is a programming mode that is aimed at very good students who are interested in discovering embedded artificial intelligence and who want to test their algorithm on a real platform.

## 4. Platform Description

**Figure 1:**
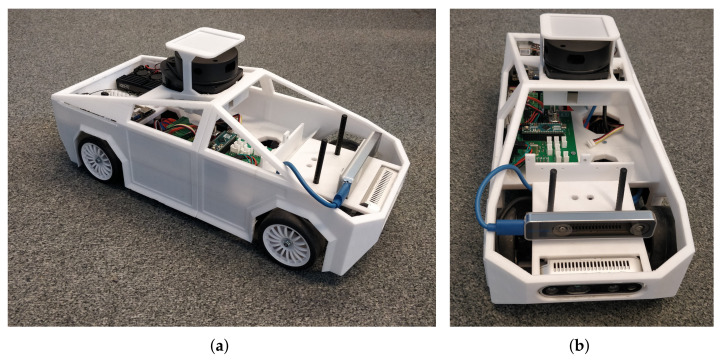
Our 1:10 platform with all of these ECUs and sensors on board. (**a**) Our 1:10 platform; (**b**) Two intel camera in the front.

We have chosen to build our platform (Figure 1) as closely as possible to the model of today’s autonomous cars. We chose to use a scale model from a well-known Tamiya brand in order to be able to easily experiment. Our model was designed to be as modular as possible, so that we could modify it according to each user’s needs. All of the developments, including the hardware and software, are available in GitHub [17,18].

### 4.1. Scale 1:10

Our model has a scale of 1:10. The car is 48 cm long and 20 cm wide, including the bodywork.

This scale seems, to us, to be the most adapted to the pedagogical objective of our platform. Indeed, it is possible to easily instrument the platform, because there is enough space to load all of the necessary components. It is possible to develop new electronic boards without having to miniaturize the board. However, it is not possible to board a complete computer on our model.

The use of a smaller scale would not have made it possible to embed modular electronics. All of the smaller robotic platforms have a single electronic board and the whole platform is static, not modular. The use of a larger scale requires access to a large room for experimentation. Indeed, the 1:10 scale makes it easy to experiment in a small space, such as a classroom of about 50 m${}^{2}$. It is quite possible to make a circuit all around the classroom to be able to experiment. In order to set up the platform, it is possible to raise it and then put it on the table to test its behavior without risking any slightest breakage.

### 4.2. Mechanics and Actuators

We chose Tamiya’s TT-02 platform. This model has a very good reputation in the modeling community. It is a very accurate representation of a car. It is controllable by the steering angle and engine speed. It features two differentials and a drive shaft. It has shock absorbers, cardan shafts, etc. Moreover, it had two main advantages for us. The first one was the possibility to have spare parts to easily repair the model, but also to upgrade some parts in order to improve its characteristics. For example, we will be able to replace the steering parts with aluminum parts to improve the car’s handling. Its low price range was the second advantage, since it only costs about 100 euros.

Our platform has two engines. There is a motor dedicated to propulsion and a servomotor dedicated to steering.

A brushed DC motor is used for the propulsion. We chose a motor with high torque and relatively low speed. Indeed, we wanted to have the highest possible maneuverability in terms of speed, while still being able to carry more weight than what is typically achieved by scale models. Therefore, we chose a 540 80T engine from GoolRC. It allows you to move easily at a low speed while carrying more weight. We also tested a Reely 511434 brushless motor (from Conrad Electronic International, Hirschau, Germany) that meets all of our needs while still allowing a very high top speed. We changed the output pinion to a pinion with fewer teeth in order to minimize the engine speed. In our case, we were able to put a 17 teeth gear. Regarding the power electronics of the engine, we chose to use a classic model controller ESC. This allows us to keep the maximum realism of the platform. In addition, these controllers are controlled with a single PWM, which is easily generated by our electronics.

We use a classic servo motor of the Futaba brand for the steering. It can be controlled by means of a PWM signal with a mid point of 1.5 ms.

### 4.3. Low-Level

**Figure 2:**
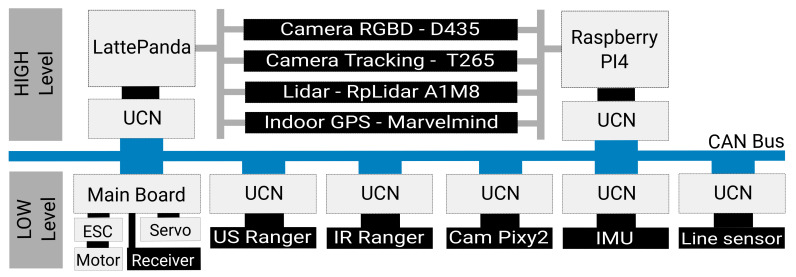
On-board scale model electronics and communication architecture.

We have seen that an autonomous car is made up of many electronic modules that are linked together by a CAN network. Therefore, we chose to use the same type of architecture for our platform (Figure 2).

#### 4.3.1. Electronics Architecture

We wanted the car’s on-board electronics to respect two main constraints: to take up the concept of modular electronics of a classic car and be easy to use and develop.

**Figure 3:**
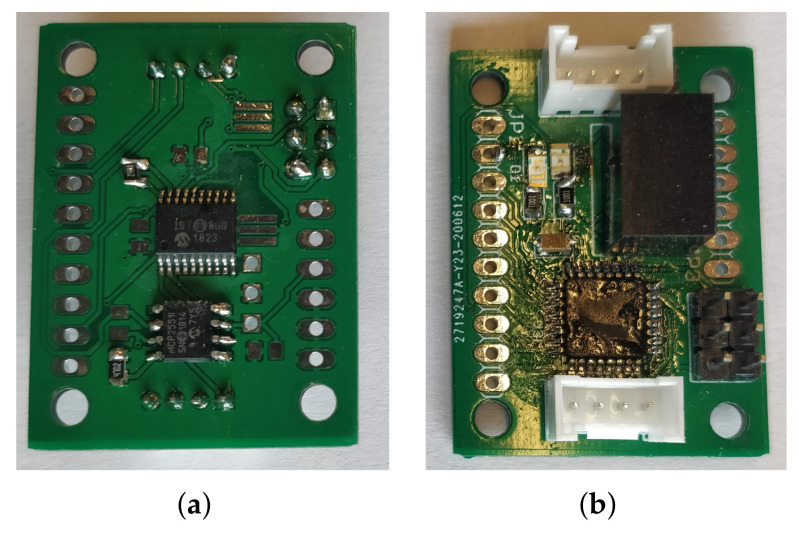
Universal CAN Node. (**a**) Rear side with CAN transceiver; (**b**) Front side with power supply and microcontroller.

Therefore, we have developed a Universal CAN Node (UCN) that is based on the Atmega328p processor (from Microchip, Chandler, AZ, USA) (Figure 3, the source file provided as additional materials). We chose this microcontroller because it is well known in the electronic community (it is the processor of several Arduino boards). There are many examples of use, and this processor has been widely tested in embedded applications. However, this processor does not have a CAN controller, so we have added an MCP2515 CAN controller (by Microchip, Chandler, AZ, USA) on the SPI bus as well as an MCP2551 CAN network driver. Both of these components are widely used in the community. Regarding the power supply of the module, we chose that each module would include a Traco DC/DC converter. Indeed, the converter is the most expensive component of our base board. The board is very small (3.6 mm × 2.6 mm), and it has four m3 mounting holes and two connectors to connect the CAN network and the power supply. The different modules can be easily linked together. We have chosen to use four-pin grove connectors from Seeedstudio. These connectors allow us to chain the modules and use cheap cables. In addition, it protects against any polarity problem.

The CAN controller also allows CAN frames to be filtered. It is necessary to filter the incoming CAN frames if you do not want to saturate the microcontrollers.

With this electronic architecture, it is possible to connect any CAN module on our platform. It is entirely possible to add existing modules or develop your own modules. Finally, if a student needs to study a particular module, then it is possible to disconnect it from the car and study it separately.

#### 4.3.2. Main Board

In addition to our basic module, we have developed a main board in order to add several functionalities to improve the handling of the car.

Our specifications for this electronic board were:

- switching between battery power and charger power;
- radio receiver signal recovery;
- control of the car possible via the microcontroller or remote control unit; and,
- CAN network interface.

The main advantage of our card is to be able to pilot our car either via a microcontroller or via a classic radio used in model making. We added a radio receiver that was used in model making. In addition, the on-board microcontroller is able to read the radio control command and control the motors. Therefore, it is possible to add on-board intelligence functions to the microcontroller.

This mode of operation is very useful for use in the car. Indeed, if we want to work on the sensors of the car, then it is not useful for it to be autonomous. On the contrary, we often want to make the car to complete very specific journeys in order to characterize the sensors. The use of the remote control makes driving the car very easy.

#### 4.3.3. Embedded Sensors

**Figure 4:**
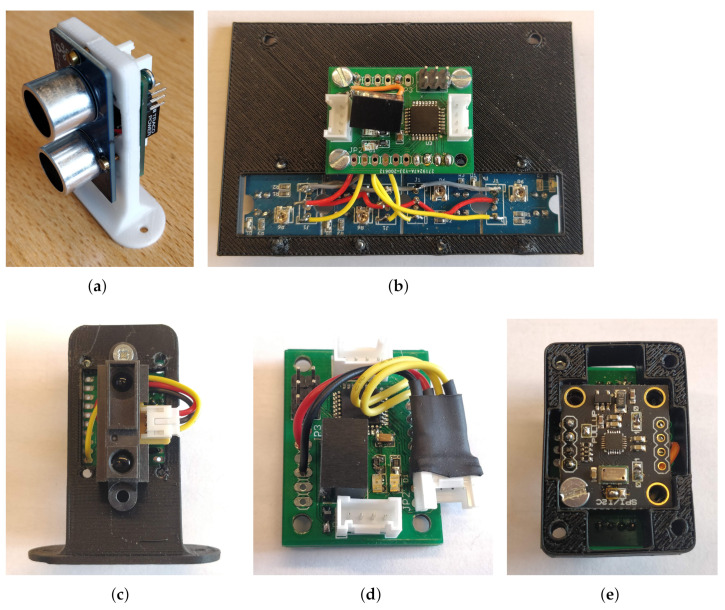
Examples of CAN node for our platform. (**a**) Ultrasonic distance sensor; (**b**) Line of ground sensors; (**c**) Infrared distance sensor; (**d**) Quadrature encoder node; (**e**) IMU node.

These perspectives will lead us to consolidate training practicals and develop model adaption studies between the scale model and a full-scale AV. On our platform, we took on board several sensors that we called low level (Figure 4). It is quite possible to equip the platform according to the objectives that we want it to achieve.

##### Inertial Measurement Unit

Our car is equipped with a nine-axis inertial unit. This sensor tells us about the rotations, accelerators, and magnetic field surrounding the car. It is an MPU9250 module that is connected to our base board. It is possible to program our card to either transmit the raw data directly to the CAN network or to pre-process the data in order to, for example, make a filter or to integrate the data as close as possible to the sensor.

##### Quadrature Encoders

When you want to use a car platform, its speed is one of the first data that you want to measure. However, it is quite complicated to find a sensor that is simple to position to measure speed. Accordingly, we used a quadrature encoder that we positioned using a 3D printed part. A gearwheel was laser cut and it is driven by the transmission shaft of the car. This sensor allows us to measure the direction of movement of the car as well as its speed. We have approximately five encoder tops per millimeter. The encoder is connected to our base board. This allows for the decoding of the quadrature signals as well as the counting of the edges as close as possible to the sensor. The counter value is then transmitted to the CAN network.

##### Ultrasonic Sensor

A first exteroceptive sensor node has been developed. It is a distance sensor using ultrasonic measurement technology. It is a sensor that is widely used in robotics. This sensor informs us regarding the presence of an obstacle in the sensor’s detection field. The sensor has been coupled to our base board in order to decode its signals and publish the results of its measurements on the CAN network.

##### Infrared Sensor

We have developed a second distance measuring node. It is an infrared distance sensor. These sensors measure the distance to the nearest object. Unlike the ultrasonic sensor, it has a narrower detection area. The sensor has been coupled to our base module in order to perform the analog acquisition and publish the results on the CAN network.

##### Line Sensor

We used a black line sensor. Indeed, one of the first missions that we try to program on a platform of this type is very often line tracking. Accordingly, we used grove-brand line sensors that we connected to our base module. It is possible to connect several sensors to one base module to limit the number of modules needed. The information is then transmitted on the CAN network.

### 4.4. High-Level

Autonomous cars carry so-called high-level sensors in order to perceive their environment. Indeed, it is necessary to know one’s environment in order to make the decisions necessary for autonomous navigation.

Therefore, we have equipped our platform with two processing units (Raspberry PI4 from Raspberry Pi Foundation, Cambridge, UK and Lattepanda) in order to be able to process the data from the so-called high-level sensors. Indeed, these sensors require high computing power to process the data.

#### 4.4.1. Embedded Computer

We have chosen to carry two high level processing units. Indeed, the two processing units offer different advantages that make it easier for students to access.

##### Raspberry PI4

A Raspberry PI4 is our first onboard computing unit. This module has several advantages. First of all, this board is very well known in the community, it supports Ubuntu, and has many drivers and resources to make it work. In addition, it has a USB3 port that allows it to take advantage of recent sensors. The Raspberry PI4 card is sufficient for processing small images in real time. If you wish to embed artificial intelligence algorithms, then it is necessary to associate it with an additional processing unit, for example, the Movidius Intel Neural Compute Stick (from Intel Corporation, Santa Clara, CA, USA).

Finally, it is possible to install ROS very easily on this card. Indeed, ROS is widely used in the robotics community and, therefore, it is important to be trained in its use.

##### Lattepanda

We wished to embed a second processing card under the MS Windows to account forf teaching in fields of study where students are not familiar with the Linux environment. We chose a LattePanda card that is usable under windows and has a USB port. For our use and our students, we used LabVIEW (from National Instruments Corporation, Austin, TX, USA) on this card to realize high level algorithms.

#### 4.4.2. Sensors

Our platform is equipped with three types of high level sensors: several cameras, a laser range finder, and an indoor GPS. These are the most commonly used sensors on autonomous cars.

##### Camera

We have the possibility to embed several cameras: a RealSense D435 or D415, a RealSense T265 from Intel Corporation, and a Pixy2 from PixyCam.

**RealSense camera D435/D415 from Intel Corporation.** Intel has been producing realsense cameras for several years. These cameras are equipped with several imagers and an infrared projector. They provide color images, but also images of the depth of the scene. The calculation of the depth maps is directly done by the camera and, therefore, does not take any computational resources. It is not necessary to make the expensive disparity map calculations while using a pair of stereoscopic cameras.

The cameras that are provided by Intel are of very good quality. The images are quite usable for on-board processing, and the chosen lenses are compatible with the processing that was usually carried out by an autonomous vehicle: the detection of markings, detection of vulnerable, detection of panels, etc. The use of these RGBD cameras allows us to design high level algorithms very quickly.

**RealSense camera T265 from Intel Corporation.** More recently, Intel has began to offer the T265 camera. This camera features two high-speed, global shutter, black and white cameras. It also includes an inertial control unit. The most interesting is that this camera embeds a Myriad coprocessor that has been programmed to realize a SLAM algorithm. Thus, this smart camera returns the position of our vehicle in its environment. It is possible to measure its displacement in three dimensions and exploit them easily. This smart camera greatly facilitates the design of high-level algorithms.

**Figure 5:**
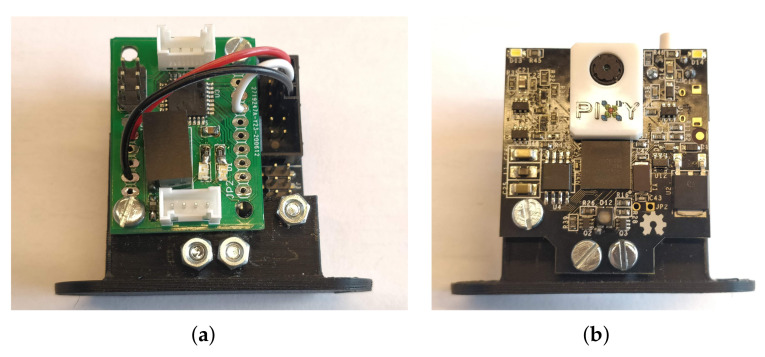
Smart camera on CAN network. (**a**) Back side; (**b**) Front side.

**Pixy 2 from PixyCam.** We have taken a lower level camera on board (Figure 5). This is the Pixy2 camera. This camera does not require an additional processing unit. It performs the processing within the camera. It then delivers the results via a serial port. Therefore, we have coupled this camera with our base board, so that we can decode the data and only publish the data that we need on the CAN network.

The purpose of this camera is not to publish images, but to publish information that is extracted from the images. For example, barcodes or lines can be detected. This type of camera enables having a knowledge of the scene and, thus, focusing on higher level algorithms.

##### Laser Scanning Rangefinder

Autonomous cars are very often equipped with a 3D laser scanner. These are mostly multi-layer rangefinders, e.g., Velodyne pucks. However, it is not possible to carry multi-layer rangefinders on our platform, because they are too large. Therefore, we have chosen to carry a single-sheet RPI LiDAR rangefinder. This rangefinder is very easy to use and it provides a point cloud at 10 Hz with a maximum range of 12 m, which is more than enough for a 1:10 scale model car moving in an indoor environment.

##### Indoor GPS

GPS is one of the most difficult sensors to reproduce for a scale model. It is not possible to use a GPS in an indoor environment. Therefore, we looked for an alternative solution to simulate a location signal in order to help navigation and reproduce the conditions of an autonomous car as well as possible. We added a Marvelmind Indoor Navigation Positioning System module (433 MHz). This module is composed of fixed beacons that are distributed throughout the room and a beacon positioned on our vehicle. This system only works if the beacons are in line of sight (like a GPS), and it allows recreating a disturbed environment scene without a location signal very easily. The system could be coupled with a base card to be available on our CAN network.

### 4.5. Programming

Several programming levels are possible. The first level is the lowest level, and it is possible to reprogram all of the basic modules. This can be done with the arduino software. Numerous examples are available for creating additional sensor nodes.

It is then possible to program the two processing units that are available on the platform: the Raspberry PI4 card and the Lattepanda card. We were able to install ROS on the Raspberry PI4 card. Indeed, many modules are available to directly interface the sensors that we have chosen: RPlidar, RealSense camera, and Marvelmind indoor localization system. ROS allows us to concentrate on the high level programming of our platform. The Lattepanda card was mainly used to create applications that are not compatible with Linux. In particular, some of our students may only have skills on Graphical programming language (e.g., LabVIEW). Hence, we were able to interface our model using a simple serial port. It is also possible to retrieve images from the cameras. Therefore, the platform is programmable at different levels and, most importantly, the programming can be adapted to different types of students.

## 5. Class Assignments

In this section, we report the experimental results in the context of teaching activities to perform with the platform. The platform was used in a project of six students for six months. The student team worked for 40 hours on the project while being supervised. They continued to work outside of class hours. They had to work on two distinct function objectives: line tracking and obstacle detection.

A general introduction of the platform is done to start working on both objectives. All of the functionalities of the platform were presented: CAN network, sensors, microcontroller, abd programming. The purpose of the first four hours session was getting started with the hardware and to better understand the project workload. Moreover, we took advantage of this session to bring them up to date with embedded programming fundamentals.

Subsequently, each student wrote the specifications that were associated with their project. These objectives will be used to evaluate their work at the end of the semester. It is worth noting that, at the beginning of the project, sthe tudents are very enthusiastic and ambitious since they are not aware of the project tasks’ complexity. Therefore, it is necessary to help them define their project.

### 5.1. Line Following

The first topic is the line following. This is a well-known topic in robotics; the original part comes from the use of nonholonomic vehicle displacement model [19] and distributed electronics architecture.

We have divided the subject into three parts:

Assignment 1: Understand the main characteristics of the sensors, program the data acquisition, and evaluate the relevance of the sensor;

Assignment 2: Send data on the CAN network and receive them on another ECU;

Assignment 3: Implement a strategy to drive the platform to follow the line.

#### 5.1.1. Assignment 1: Sensors

Our students have chosen to use a line detector composed of five infrared detectors that are disposed in a row. Their numerical output indicates the presence/absence of a reactive surface (i.e., line). They added an MBED microcontroller (F303K8) (from STMicroelectronics, Geneva, Switzerland) which performs a periodic acquisition of sensor measurements.

In the case of line sensors, the sensor evaluation was relatively straightforward. They only need to verify that the sensor detects the right color, regardless of the outdoor lighting. There is no variation in detection, because the sensor is very close to the ground.

#### 5.1.2. Assignment 2: Can Network

They added a CAN transceiver (MCP2551 from Microchip, Chandler, AZ, USA) to send the data on the CAN network. During their first test, they sent each data as a byte. Messages are then constrained to be formatted in binary in order to optimize the performances. The five sensor states can be transmitted into a single eight-bit word.

#### 5.1.3. Assignment 3: Strategy

Based on the line detector measurements, a simple algorithm infers the correct steering angle for lane keeping. Subsequently, the command is sent over CAN network.

At the end of the semester, we have added a new constraint. The width of the line was variable and it was employed to control longitudinal speed (i.e electric throttle). That is, the larger the line, the slower the speed of the vehicle. Therefore, the students had to modify their algorithm to take the new behavior into account.

#### 5.1.4. Feedback on the Assignment

Through this project, the students became familiar with the use of a microcontroller and the CAN network. They faced some difficulties while taking the latency due to the data transmission into account. Indeed, this type of servoing requires an important update frequency, and the CAN network, in some cases, limits the performance of the system.

If we had more time, then we would have asked them to remove the CAN network to compare the performances when using a single microcontroller.

### 5.2. Obstacle Detection

The second topic is the obstacle detection. This is an essential function of an AV. Regarding the first topic, we have structured the objective following three assignments:

Assignment 1: Understand the main characteristics of the sensors, program the data acquisition, and evaluate the relevance of the sensor;

Assignment 2: Send data on the CAN network and receive them on another ECU;

Assignment 3: Implement a strategy to stop the platform in front of an obstacle.

#### 5.2.1. Assignment 1: Sensors

Two sensing technologies were studied for obstacle detection: ultrasound and infrared range finders. Both of the technologies provide different advantages and drawbacks. The ultrasound range detectors were selected, since they cover a bigger field of view. To detect obstacles in front of and behind the vehicle, two sets of three ultrasound detectors were installed in the car’s bumpers. Because ultrasound range is obtained from a time-of-flight measure, its operation requires a sequential acquisition (synchronization) to avoid mutual interference between the sensors. This constraint was integrated on the micro-controller (F303k8) as well as the sensor’s calibration. The students were then able to calibrate their sensors.

#### 5.2.2. Assignment 2: CAN Network

Regarding the first group, the students added a CAN transceiver to send data on the platform network. They chose to send the six measurements on a single CAN frame. Each measure is transmitted in centimeters units.

#### 5.2.3. Assignment 3: Strategy

**Figure 6:**
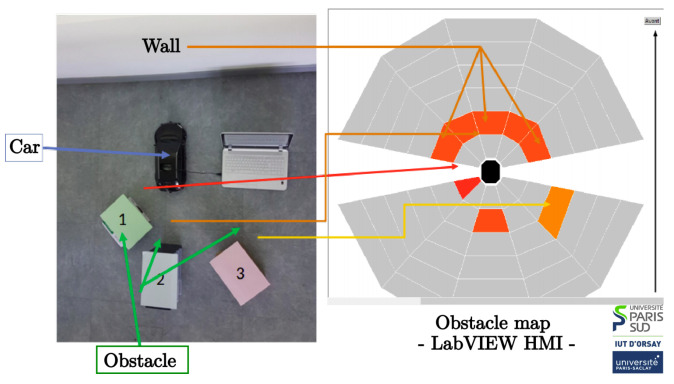
Obstacles map.

Obstacle detection results are represented in a discrete map of the vehicle surroundings (see Figure 6) on the main processing unit. In the presence of an obstacle that is close to the vehicle, an emergency braking message is emitted to set the vehicle speed to zero. This command is taken into account by the electronic board that controls the two actuators of the car.

#### 5.2.4. Feedback on the Assignment

The students were able to apply the skills they have learned in sensors and embedded electronics. They were able to set up a real experiment that highlights problems that one can encounter in real life. They were confronted with problems that are related to acquisition time, latency, and data formatting. Even if their final application results are not perfect, the pedagogical goal is still fully met.

## 6. Experimental Platform Concept Validation

Beyond our first tests with students, we wanted to test the platform in greater depth by performing more complicated experiments. Such a test is intended to provide a concept proof that experimentally validates the didactic model concept and it confirms the transposability of fundamental state-of-art perception algorithms.

The experiment was conducted in an indoor environment where the scale-model follows a 20 m-trajectory at low speed (0.13 ms${}^{-1}$ on average). During this experience, the embedded computer records on-the-fly asynchronous LiDAR scans at 7 Hz. The collected data were recorded in a time-associated ROSbag file that is publicly available at [18]. Two state-of-the-art perception algorithms were selected to evaluate the data provided by the introduced platform: a LiDAR-based SLAM and a Computer Vision-based object detector. The proposed algorithms were considered to be key representative perception applications for autonomous navigation tasks. That is, because autonomous vehicles must be able to detect surrounding objects, perform self-localization and mapping. In the following, the obtained results are reported and analyzed.

### 6.1. Lidar-Based Simultaneous Localization and Mapping

LiDAR-based SLAM implementation that was inspired from Hess et al. [20] was employed to perform LiDAR scan matching, motion estimation, and mapping. This approach is based on a classical scan matching process that estimates the relative motion of the scale-model vehicle from current and previous LiDAR measurements. Subsequently, a grid-based environment representation is updated and enriched with recent LiDAR data. In particular, the cited approach excels with a good performance thanks to a regular pose optimization that takes advantage of all completed sub-maps of the environment global map for enforcing the loop-closure.

**Figure 7:**
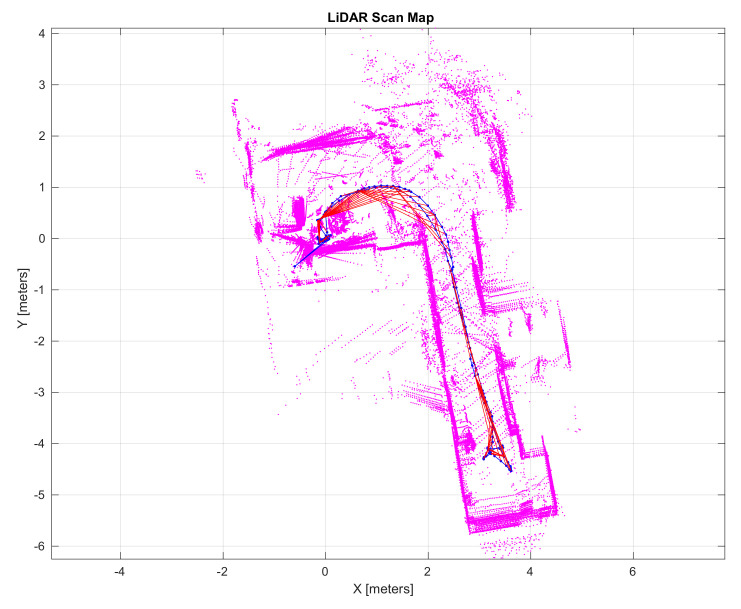
LiDAR-based SLAM results.

In our experiments, the trajectory that is followed by the model starts with a slight right turn, and the vehicle then evolves straight along an aisle and finally turns and comes back close to the starting point of the trajectory, as illustrated in blue in Figure 7. Multiple geometrical constraints (red segments in Figure 7) on the trajectory were automatically inferred by the SLAM algorithm because the trajectory followed by the model describes a loop. Those constraints allow a graph pose optimization for reducing the scan misalignment and localization errors.

**Figure 8:**
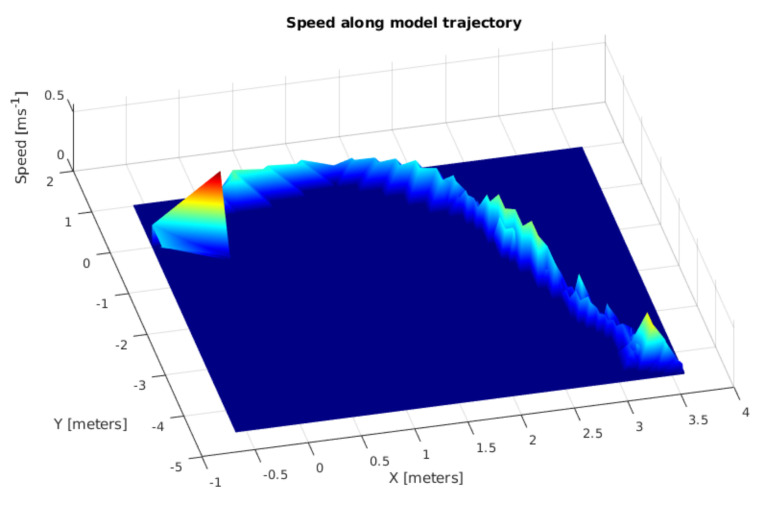
Speed profile of the scale-model vehicle along the trajectory.

When considering the estimated SLAM trajectory, Figure 8 illustrates, along with the z-axis speed, changes of the scale-model vehicle during the experiment. This figure also highlights important speed changes while the model starts moving; such changes happen at the beginning of the test and when the model changes direction and comes back to the starting point.

**Figure 9:**
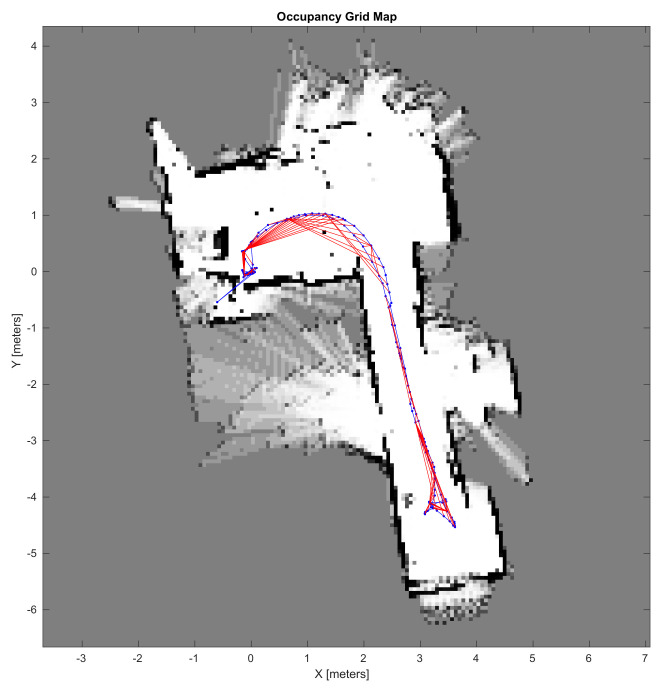
Mapping results—Occupancy grid representation.

The LiDAR scan map that is illustrated in Figure 7 integrates scan measures, including static and moving objects that are situated in the vehicle surroundings. This environment representation is certainly not suitable for navigation algorithms to efficiently perform. With the aim of inferring the static structure of the environment, an occupancy-grid based analysis was carried out. Figure 9 excels the obtained results in a 10 × 10 m grid representation at 5 cm resolution. White cells stand for map regions that are free of obstacles, gray cells represent unexplored regions where a few or no measures were collected, and black cells identify highly probable map regions where obstacles are situated.

### 6.2. Computer Vision-Based Object Detector

Semantic scene understanding is a key step towards highly automated navigation systems. This concept was applied to the proposed platform by deploying an object detector pre-trained Deep Neural Network (DNN) model [21] on the embedded computer. This selected approach performs object detection by globally classifying images from features and predicting object bounding boxes from the confidence class levels. Its high computational efficiency and good detection performance make this algorithm a very interesting approach for the scale-model vehicle. The images from an onboard RGBD camera were inputted to the neural network through a use-case scenario where three scale-models are observed while the vehicle moves.

**Figure 10:**
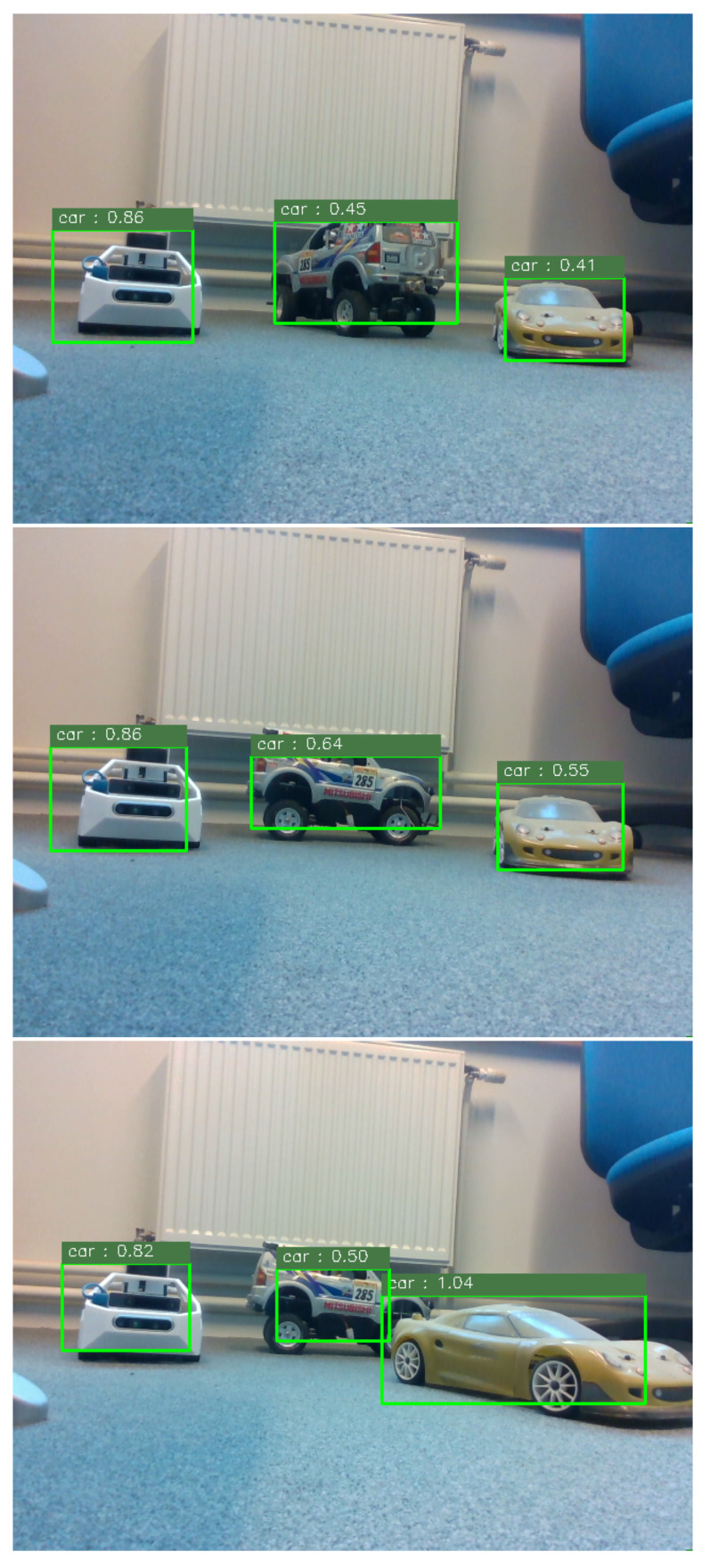
The object detector results deploying a pre-trained neural network model on-board the proposed scale-model vehicle prototype.

Figure 10 illustrates the object detection reports predicted from the pre-trained model and overlayed to the corresponding processed images. Based on the obtained results, the transposability of such object detection approaches was confirmed to be well-suited and robust to scale and orientation changes. However, it is worth noting that the confidence accorded to the detected object remains sensible while detecting scale-models that do not follow a classical car-like appearance or that are partially occluded.

### 6.3. Discussion and Analysis

Through the proposed experiments and reported results, our scale-model vehicle has proved to be a reliable, flexible, and well-suited experimental learning platform. These experiments are considerably more complex than those that were performed by our students. We think that they can be realized by students of a Master level.

Its perception capabilities allow for the development and deployment of elegant state-of-the-art perception methods. To this end, a LiDAR SLAM and a DNN-based object detector were deployed, retrieving satisfactory results addressing self-localization, mapping, and semantic scene analysis. It is worth noting that both of the evaluated algorithms rely on single data sources. However, any asynchronous data fusion strategy will be suitable when considering the fact all sensor data are locally referenced to a common timing system.

## 7. Perspectives

Currently, our platform is complete and fully functional. We wish to develop two axes of perspectives.

The first axis concerns communication between several vehicles and between a vehicle and its environment. For the moment, our vehicle is equipped with all of the sensors that are necessary for the perception of its environment. However, it does not have radio communication, which allows for data exchange between vehicles. The simplest solution would be to use the WIFI communication that is available on the Raspberry PI4 card which is already on board our model. However, the use of WIFI seems to be unrealistic in the context of autonomous vehicles. Therefore, a radio module will have to be developed and interfaced to our CAN bus. It will allow V2V and V2X communication.

The second axis concerns the experimentation environment. We are currently using a simple classroom without any modification. Therefore, the size of the objects is not reduced to the 1:10 scale, and this may lead to bias in the perception algorithms. Therefore, we wish to create an environment on the scale of our platform and to be as realistic as possible visually.

These perspectives will lead us to consolidate training practicals and develop model adaption studies between the scale-model and full-scale autonomous vehicle.

## 8. Conclusions

The use of a real scale vehicle is very complicated in teaching. Its cost is very high and its experimentation is complicated in optimal conditions because of the space that is required, but also because of safety constraints. Therefore, we have designed a platform that is dedicated to the learning of technology that is embedded in autonomous vehicles. It can be associated with different learning levels from low level electronics to high level learning algorithms. It embeds all of the components of a current autonomous car. Moreover, it is very modular and can be modified according to the needs of teachers.

## Figures and Tables

**Table 1:** Robotics and scale vehicle-like platforms summary.

Robot Platform	Size	Motion Model	Electronics Architecture	Perception Sensors	Onboard Processing
[4] Epuck	70 mm	Differential	Centralized	3D accelerometer, infrared distance, camera, microphones, range and bearing turret, omni-directional camera	dsPIC 30F
[5] Pheeno	10 cm	Differential	Centralized	3D accelerometer, 3D magnetometer, wheel encoder, infrared distance, camera	ATmega328P, Cortex-A7
[6,7] Thymio	11 cm	Differential	Centralized	3D accelerometer, infrared distance, microphone, temperature, touch	PIC24F
[8] MarXbot	17 cm	Differential	Distributed	3D accelerometer, 3D gyrometer, infrared distance, omni-directional camera, front camera, RFID, 2D force	ARM11, x*dsPIC
[9] Mindstorm		Differential	Centralized	gyroscope, infrared distance, color, touch, ultrasonic	TI Sitara AM1808
[10] IRobot Create	34 cm	Differential	Centralized	encoders, wall, infrared distance, Cliff sensors	
[11] Kilobot	4 cm	Vibration motors	Centralized	infrared distance, ambient light	Atmega328
[12] Zooids	2.6 cm	Differential	Centralized	touch sensor	STM32F
[13] MIT Racecar	1/10e	Car model	Centralized	bumper, laser scanner, RGBD camera	Nvidia Jetson Nano
[14] AWS DeepRacer	1/18e	Car model	Centralized	3D accelerometer, 3D gyrometer stereo camera, laser scanner	Intel Atom
[15] Quanser QCar	1/10e	Car model	Centralized	3D accelerometer, 3D gyrometer, 3D magnetometer, encoder, laser scanner, RGDB camera, 360 camera, microphone	Nvidia Jetson TX2
[16] F1TENTH	1/10e	Car model	Centralized	3D accelerometer, 3D gyrometer, 3D magnetometer, laser scanner, RGBD camera	Nvidia Jetson TX2
Our Platform	1/10e	Car model	Distributed	3D accelerometer, 3D gyrometer, 3D magnetometer, US distance, encoder laser scanner, RGBD camera, Indoor GPS	Raspberry PI4, LattePanda, x*Atmega328

## Data Availability

Dataset - Scaled-model vehicle - Paris-Saclay University (2020-06-04-00-22-50) https://doi.org/10.17632/6xs7pzpmz6.1 (accessed on 31 May 2021), AutonomousVehicle. https://github.com/BastienV-SATIE/AutonomousCar/ (accessed on 31 May 2021).
